# Treatment of extended-spectrum β-lactamase-producing *Enterobacteriaceae *(ESBLs) infections: what have we learned until now?

**DOI:** 10.12688/f1000research.14822.1

**Published:** 2018-08-29

**Authors:** Zoi Dorothea Pana, Theoklis Zaoutis

**Affiliations:** 1 Infectious Diseases Department, 3rd Department of Pediatrics, Hippokration General Hospital Aristotle University, Thessaloniki, Greece; 2 Infectious Diseases Department, The Children’s Hospital of Philadelphia, Perelman School of Medicine at the University of Pennsylvania, Philadelphia, PA, USA

**Keywords:** lactamase producers, ESBL treatment, Enterobacteriaceae

## Abstract

The spread of extended-spectrum β-lactamase (ESBL)-producing *Enterobacteriaceae* (ESBL-PE) has dramatically increased worldwide, and this “evolving crisis” is currently regarded as one of the most important public health threats. The growing problem of ESBL-PE antimicrobial resistance seems to have a dual face between “Scylla and Charybdis”: on one hand the potential for rapid spread and dissemination of resistance mechanisms and on the other hand the injudicious overuse of antimicrobial agents and the inadequate infection control measures, especially in the health-care setting. Given the World Health Organization’s warning against a “post antibiotic era”, health-care providers are at a critical standpoint to find a “balance” between safe and effective ESBL-PE treatment and avoidance of inducing further resistance mechanisms. The aim of the review is to summarize the updated published knowledge in an attempt to answer basic everyday clinical questions on how to proceed to effective and the best ESBL-PE treatment options based on the existing published data.

## Introduction

Extended-spectrum β-lactamase (ESBL) enzymes are characterized by the ability to hydrolyze third-generation cephalosporins and aztreonam but are inhibited by clavulanic acid. The spread of ESBL-producing *Enterobacteriaceae* (ESBL-PE) has dramatically increased worldwide, and this “evolving crisis” is currently regarded as one of the most important public health threats. The growing problem of ESBL-PE antimicrobial resistance seems to have a dual face between “Scylla and Charybdis”: on one hand the potential for rapid spread and dissemination of resistance mechanisms and on the other hand the injudicious overuse of antimicrobial agents and the inadequate infection control measures, especially in the health-care setting. A multicenter study in the US reported that in 2012 the prevalence of ESBL-producing *Klebsiella pneumoniae* reached 16% and almost 12% for ESBL-producing *Escherichia coli* and that rates were much higher among intensive care unit (ICU) patients ^1^. Even in the pediatric population, a meta-analysis revealed that the worldwide prevalence of ESBL producers was estimated to be 9% (11% neonates and 5% children) with an annual increase of 3.2% and a wide variability among different geographic regions (15% in Africa, 12% in South America, 11% in India, 7% in the rest of Asia, and 4% in Europe) ^2^.

ESBL-PE are associated with increased morbidity and mortality rates, prolonged hospital stays, and increased costs. In a matched cohort study, the nosocomial financial burden of non-urinary tract infections (non-UTIs) caused by ESBL producers was 1.7 times higher compared with the same type of infections caused by non-ESBL producers ^3^. A case control study in Canada showed that ESBL-PE infections were significantly associated with increased cost (C$10,507 versus C$7,882), hospitalization (8 versus 6 days), and mortality rates (17% versus 5%) ^4^. In addition, data regarding the rates of ESBL-PE colonization (both health-care or community acquired) reveal an increasing trend over time with significant differences among several geographical regions and patient groups ^5^. For high-risk patients in the ICU, the ESBL-PE colonization rates might range from 2.3% for the US to 49% for India. According to a recently published systematic review, the most frequently reported risk factor for ESBL-PE colonization and infection remains prior exposure to antimicrobials as well as recent hospitalization and recent or repeated surgery ^5^. Although prior ESBL-PE colonization has been shown in a few studies to increase the risk of acquiring an ESBL-PE infection, further data are needed.

Given the World Health Organization (WHO) warning against a “post antibiotic era”, health-care providers are at a critical standpoint to find a “balance” between safe and effective ESBL-PE treatment and avoidance of inducing further resistance mechanisms. The aim of this review is to summarize the updated published knowledge in an attempt to answer basic everyday clinical questions on how to proceed to effective and the best ESBL-PE treatment options based on the existing published data.

## Before starting ESBL treatment

The first step before initiating ESBL-PE antimicrobial treatment is to carefully evaluate specific parameters that are directly associated with ESBL-PE therapeutic decision making. Of utmost importance is to clearly characterize (a) the isolate with the *in vitro* susceptibilities, (b) the location of the infection, (c) the degree of source control of the infection, and finally (d) the clinical condition of the patient ( Table 1). In addition, recently published studies propose that all ESBL-PE do not belong in the same homogenous group as far as comorbidities, presentation, and outcome are concerned ^6^. In particular, data have shown that bloodstream infections (BSIs) associated with ESBL-producing *E. coli* were more frequently of a urinary source and community onset compared with BSIs with ESBL-producing *Klebsiella* spp. ^6^.

**Table 1.** Significant parameters for extended-spectrum β-lactamase-producing Enterobacteriaceae (ESBL-PE) antimicrobial treatment.

Infection’s location	High- ^a^ versus low-inoculum infection
Infection source control	Adequate or no source control
Patient’s clinical condition	Critically ill patient; presence of immunosuppression
Characterization of multidrug-resistant Gram-negative *Enterobacteriaceae*	Mechanisms associated with ESBL, AmpC, or carbapenem-resistant Enterobacteriaceae
Evaluation of minimum inhibitory concentration (MIC) susceptibilities	Especially for carbapenems, cefepime, and β-lactam/β-lactamase inhibitor (BLBLI) combinations
Type of ESBL-PE	*Klebsiella pneumoniae* versus *Escherichia coli*

^a^Examples of high-inoculum infections could be large intra-abdominal collections and endocarditis vegetations.

## Decision making on ESBL-PE antimicrobial treatment

### Clinical question 1: Carbapenems *or* β-lactam/β-lactamase inhibitor combinations in ESBL-PE infections?

Carbapenems possess the broadest spectrum of β-lactam antibiotics with greatest potency against Gram-negative bacteria and are characterized by stability to hydrolysis by the majority of β-lactamases ^7^. Several studies have shown that carbapenem treatment is associated with improved outcomes in patients with severe ESBL-PE infections and remains the “gold standard” treatment for serious and invasive ESBL-PE infections ^8, 9^. Specific considerations among carbapenems are the induction of carbapenem resistance and their side effects, especially as far as their epileptogenic effect is concerned ^10^. Most studies have evaluated either meropenem or imipenem for the treatment of ESBL-PE, although a recently published multinational retrospective study compared the clinical efficacy of ertapenem with that of other carbapenems in ESBL-PE BSIs ^11^. Cure rates were similar (90.6% with ertapenem and 75.5% with other carbapenems in empiric and 89.8% and 82.6% in targeted treatment), and no differences have been observed for mortality among the two groups, but for patients with severe sepsis a non-significant trend favoring other carbapenems was observed ^11^.

Among β-lactam/β-lactamase inhibitor (BLBLI) combinations, the combination of piperacillin–tazobactam (PTZ) has been extensively studied as an alternative carbapenem-sparing option against ESBL-PE infections ^12^. In 2012, a meta-analysis compared the mortality rates among carbapenems and alternative regimens, including BLBLIs, for the treatment of ESBL-PE BSIs ^13^. According to their results, differences were noticed in mortality rates when administered as either definitive or empirical therapy, although they mentioned one study’s significant heterogeneity ^13^. Since the question about the role of BLBLIs remained, six subsequent studies from 2012 to 2017 tried to elucidate the role of BLBLIs against ESBL-PE with rather conflicting results ^14– 18^. These controversies were interpreted by substantial differences among the studies’ design. In particular, the Spanish groups included mainly *E. coli* as the attributed ESBL-PE, and their studies had lower inoculum infections, ICU admissions, and median PTZ minimum inhibitory concentration (MIC) and higher PTZ treatment dosages compared with the study by Tamma *et al*. ^9, 15, 18^. A further analysis of cases of the Spanish group, conducted by Retamar *et al*., revealed that all patients who presented with ESBL-PE BSIs from a urinary source had a favorable outcome, irrespectively of the PTZ MIC ^19^. Furthermore, among patients with an ESBL-PE BSI with a source other than a urinary one, the outcome was dismal when the MIC of the isolate for PTZ was more than 2 mg/L ^19^. A recently published randomized controlled trial was conducted comparing PTZ, cefepime, and ertapenem for the treatment of ESBL-PE UTIs caused by *E. coli* ^20^. Based on the results, the clinical and microbiological response to PTZ treatment was estimated to be 94% and was similar to the response to ertapenem treatment. An ongoing retrospective observational study (BICAR) is trying to evaluate the efficacy of BLBLI combinations for the treatment of ESBL-PE BSIs in hematological patients with neutropenia ^21^. In addition, a recently published propensity score-weighted multicenter cohort study in Korea showed that, among 232 patients with ESBL-PE BSIs, non-carbapenem regimens were not inferior to carbapenems (30-day mortality rates for non-carbapenems 6.3% versus carbapenems 11.4%) ^22^.

***Authors’ recommendations.*** For invasive, high-inoculum ESBL-PE infections with a source of infection other than *E. coli* and in critically ill patients, carbapenems remain the “gold standard” of targeted treatment. Especially for ICU patients, according to a recent systematic review, the empirical use of PTZ when high risk of ESBL-PE is suspected should be avoided ^23^. Definitive therapy with BLBLIs should be selected under specific criteria such as stable condition, after microbial documentation with susceptibility results, in combination with dose and infusion modalities to the MIC in order to reach pharmacological targets ^23^.

Definite answers concerning the role of BLBLIs (and specifically PTZ) against ESBL-PE BSIs will be given by an ongoing multicenter clinical trial (MERINO trial) comparing PTZ versus carbapenems for the definitive treatment of BSIs caused by ceftriaxone-resistant *E. coli* and *Klebsiella* spp. ^24^. Based on the preliminary MERINO results presented at European Congress of Clinical Microbiology & Infectious Diseases 2018, the most common ESBL-PE bacteremia source was the urinary tract (60.9%) with clear predominance of *E. coli* (86.5%). Although no difference between the two groups regarding subsequent infections of drug-resistant bacteria or *C. difficile* was reported, the 30-day mortality rate differed (12.3% for PTZ versus 3.7% for meropenem) ^25^. In addition, it is of utmost importance to clearly define in future studies the specific subset of patients with ESBL-PE infections who could benefit from carbapenem-sparing treatments, especially regarding hematological patients with neutropenia ^26^.

### Clinical question 2: What is the role of cefepime in treating ESBL-PE infections?

The results from published studies and reviews evaluating the efficacy of cefepime versus carbapenems for the treatment of ESBL-PE infections remain controversial. Few studies have shown comparable efficacy, whereas others reported significant inferiority of cefepime ^27– 31^. Among these studies, Lee *et al.* ^31^ and Wang *et al.* ^27^ showed significantly lower mortality rates at 30 and 14 days, respectively (17% versus 59% and 41% versus 20%, respectively). In particular, in the study by Lee *et al*., a significant association was observed between the mortality rates of the patients receiving cefepime and the MIC of the drug. In particular, for cefepime MIC of not more than 1 μg/mL, the mortality rate was 16.7%; for MIC of 2–8 μg/mL, the rates reached 45.5%; while for MIC of at least 16 μg/mL, the rates were 100% ( *p* = 0.035) ^31^. Even after propensity score adjustment, cefepime remained inferior compared with ** carbapenem (adjusted odds ratio 6.8, 95% confidence interval (CI) 1.5–31.2, *p* = 0.01). A subsequent randomized controlled trial was conducted comparing PTZ, cefepime, and ertapenem for the treatment of ESBL-PE UTIs caused by *E. coli* showing inferiority of cefepime compared with the other treatment options. The efficacy of cefepime was 33.3% compared with 94% efficacy of PTZ treatment ^20^.

***Authors’ recommendations.*** For serious invasive ESBL-PE infections with high inoculum and lack of source control, cefepime seems to be inferior compared with carbapenems because of two significant issues: increased MICs of the drug because of high inoculum effect and failure to achieve its pharmacodynamic targets in severe ESBL-PE infections. Cefepime could be administered only in non-severe ESBL-PE UTIs, where high drug concentrations could be achieved and when simultaneously low MIC of the drug is reported (MICs ≤2 μg/mL).

### Clinical question 3: What is the role for fosfomycin, aminoglycosides, or temocillin in ESBL-PE infections?

Fosfomycin is an old bactericidal antibiotic agent (phosphonic compound) with a unique mode of action of inhibiting bacterial cell wall biosynthesis ^32, 33^. A recently published literature review concerning the susceptibility of contemporary Gram-negative bacteria revealed that, for ESBL-producing *E. coli*, susceptibilities ranged from 81% to 100% and, for ESBL-producing *K. pneumoniae*, from 15% to 100% ^34^. Owing to its low molecular weight, its hydrophilicity, and its negligible serum protein binding, the drug achieves good tissue penetration and high concentrations in the serum, soft tissue, lungs, bone, cerebrospinal fluid, and heart valves ^35, 36^. Especially for the urinary tract, the drug achieves high concentrations for a prolonged period of time. Finally, in critically ill patients, a significant increase of fosfomycin volume of distribution is observed; therefore, the current paucity of data on fosfomycin in critically ill patients prevents accurate dosing guidance ^36^. Clinical data concerning the efficacy of intravenous fosfomycin against ESBL-PE invasive infections are very limited and focus mainly on UTI treatment ^32, 37– 42^. A randomized clinical trial (“FOREST”) comparing the safety and efficacy of fosfomycin versus meropenem in bacteremic UTIs caused by ESBL-producing *E. coli* is ongoing ^38^. Fosfomycin as monotherapy for the treatment of multidrug-resistant organism (MDRO)-associated invasive infections is limited by the emergence of drug resistance to fosfomycin during treatment ^39^. A more recently published meta-analysis conducted by Grabein *et al.* tried to summarize the current clinical evidence of intravenous fosfomycin in 128 studies ^43^. According to their results, the drug showed comparable clinical or microbiological efficacy compared with other antibiotics when used for sepsis/bacteremia, urinary tract, respiratory tract, bone and joint, and central nervous system infections ^43^. The pooled estimate for resistance development during fosfomycin monotherapy was 3.4% (95% CI 1.8%–5.1%). ****

Up-to-date data concerning the role of aminoglycosides in combating MDRO infections showed that for ESBL infections they can be used as part of a combination regimen, especially for UTIs and intra-abdominal infections (IAIs), as a carbapenem-sparing option ^8^. An *in vitro* synergistic effect has been confirmed for the concomitant administration of aminoglycosides plus β-lactams, while the monotherapy is not generally recommended for ESBL-PE infections, except for ESBL-PE non-bacteremic UTIs, mainly owing to the high risk of resistance development ^44– 47^. Among newer aminoglycosides, plazomicin (formerly ACHN-490), a next-generation aminoglycoside synthetically derived from sisomicin, is recently gaining more attention against MDRO infections ^47, 48^. The unique characteristic of plazomicin is its resistance to inactivation by aminoglycoside-modifying enzymes compared with other agents of the same group ^47, 48^. However, plazomicin is not active against bacterial isolates expressing ribosomal methyltransferases ^47, 48^. In two studies, plazomicin has been shown to be more potent than other aminoglycosides in treating *Enterobacteriaceae* ^49, 50^.

Temocillin is a β-lactamase-resistant carboxypenicillin active against both ESBL-PE and AmpC-producing *Enterobacteriaceae* and with limited activity against *Pseudomonas*, *Acinetobacter* spp., and anaerobic bacteria. Although this carbapenem-sparing drug option is licensed in only a few European countries (UK and Belgium), data from a multicenter study in the UK among 92 infection episodes (42 BSIs) treated with temocillin showed promising results ^51^. In particular, both clinical and microbiological cure rates were reported to be 86% and 84% ^51^. In addition, a prospective randomized controlled trial conducted in Belgium in 2014 showed that for critically ill patients the optimal dose regimen for temocillin in order to achieve its pharmacological targets with longer free-serum concentrations is 2 g three times a day administered by continuous infusion ^52^.

***Authors’ recommendations.*** Fosfomycin is strongly suggested for ESBL-PE UTIs and as a step-down therapy in source-controlled ESBL-PE infections. A randomized clinical trial (“FOREST”) comparing the safety and efficacy of fosfomycin versus meropenem in bacteremic UTIs caused by ESBL-producing *E. coli* is ongoing ^41^. Other options of source-controlled ESBL-PE UTIs are aminoglycosides, especially for cystitis infections. In addition, they can be used as part of a combination regimen, especially for UTIs and IAIs, as a carbapenem-sparing option ^8^. For temocillin, larger clinical studies among different patient groups are needed in order to establish their role as a valuable carbapenem-sparing option against ESBL-PE BSIs.

### Clinical question 4: What is the role of the newly approved drugs against ESBL-PE infections?

In Table 2, some of the new drugs active against multidrug-resistant bacteria, including ESBL-producing ones, are reported. Among newer BLBLIs developed, two of them—ceftazidime–avibactam and ceftolozane–tazobactam—have already received US Food and Drug Administration (FDA) approval and therefore will be discussed further.

**Table 2.** New drugs with *in vitro* activity against extended-spectrum β-lactamase-producing Enterobacteriaceae (ESBL-PE).

New drug	*In vitro* activity
Ceftazidime–avibactam	**ESBL** AmpC *Klebsiella pneumoniae* carbapenemase (KPC) OXA-48 Not active against metallo-β-lactamase (MBL)
Ceftaroline–avibactam	**ESBL** Methicillin-resistant *Staphylococcus aureus* AmpC KPC OXA-48?
Ceftolozane–tazobactam	**ESBL** Some AmpC Multidrug-resistant *Pseudomonas aeruginosa*
Imipenem–relebactam	**ESBL** AmpC KPC OXA-48 Not active against MBL
Plazomicin	**ESBL** AmpC KPC OXA VIM Not active against some NDM

Ceftazidime–avibactam is a combination of cephalosporin with a new non-BLBLI that is generally active against *Enterobacteriaceae* and *P. aeruginosa* producing class A β-lactamases (ESBLs and KPCs) and class C β-lactamases (AmpCs) and some *Enterobacteriaceae* producing class D β-lactamases (OXAs) but lacks activity against class B carbapenemases and is less active against anaerobes compared with other BLBLIs. A phase 3 trial (RECLAIM 1 and RECLAIM 2) conducted by Mazuski *et al.* evaluated the efficacy of ceftazidime–avibactam in treating complicated IAI (cIAI), revealing non-inferiority of the tested combination drug and similar clinical cure rates of 81.6% versus 85.1%, respectively ^53^. A subsequent phase 3 (REPRISE) study by Carmeli *et al.* recently published the results of the efficacy of ceftazidime–avibactam—2 to 0.5 g intravenously every 8 hours (q8h)—versus the best available therapy both for complicated UTI or cIAI due to ceftazidime-resistant *Enterobacteriaceae* or *P. aeruginosa* with similar clinical cure rates ^54^. Finally, in 2015, the drug was approved by the FDA for complicated UTIs and cIAI with a recommended dosage of 2 g/0.5 g) 8 hourly for 7 days for UTIs and 4 to 14 days for IAIs with dose adjustment in renal insufficiency. An ongoing clinical trial is evaluating the safety and efficacy profile of the drug for nosocomial pneumonia ^55^.

Ceftolozane–tazobactam is a co-formulation of a novel cephalosporin with an old β-lactamase inhibitor. Ceftolozane is a new cephalosporin based on the scaffold of ceftazidime—with only one modification of the side chain at the 3-position of the cephem nucleus—with improved activity against multidrug-resistant *Pseudomonas* spp. Ceftolozane, like other oxyimino-cephalosporins such as ceftazidime and ceftriaxone, is not stable against class A, B, or D β-lactamases (mainly ESBLs or carbapenemases). The combination with tazobactam significantly broadens its spectrum against ESBL-PE and against few anaerobes ^56, 57^. In 2014, the FDA approved the administration of the combination drug for the treatment of complicated UTIs and cIAIs based on previously published clinical trials (ASPECT trials) ^58– 60^. In particular, the drug was evaluated in phase 3 non-inferiority clinical trials versus levofloxacin 750 mg daily in complicated UTI or meropenem 1 g q8h in cIAI. The UTI trial compared ceftolozane 1,000 mg q8h versus ceftazidime 1,000 mg q8h, including pyelonephritis, and demonstrated similar microbiologic and clinical outcomes, as well as a similar incidence of adverse effects after 7 to 10 days of treatment, respectively. The second cIAI trial has been conducted comparing ceftolozane–tazobactam 1,000/500 mg and metronidazole 500 mg q8h versus meropenem 1,000 mg q8h in the treatment of cIAI. The recommended FDA dosage is 1 g/0.5 g 8 hourly for 7 days in complicated UTIs and 4 to 14 days in cIAIs, respectively ^61^.
